# 983 Revised TBSA Utilizing a Provider Champion

**DOI:** 10.1093/jbcr/iraf019.514

**Published:** 2025-04-01

**Authors:** Nicole Kopari, Cambria Coffman

**Affiliations:** UCSF Fresno Leon S. Peters Burn Center; UCSF Fresno Leon S. Peters Burn Center

## Abstract

**Introduction:**

Accurate determination of total body surface area (TBSA) is essential in the management of burn patients, influencing fluid resuscitation, level of care, treatment strategies, and prognostic outcomes. The Lund-Browder chart is a commonly used tool to estimate TBSA, but variabilities in accuracy arise depending on the experience of the provider filling out the chart. We sought to identify if a provider champion improves the accuracy of the TBSA resulting in changes in medical management improving patient outcomes.

**Methods:**

A retrospective chart review was conducted on all admitted burn patients from January 1, 2024 to September 1, 2024 to an ABA verified burn center. The initial TBSA from the Lund Browder chart was compared to the revised TBSA which was performed within the first 72 hours of admission by a provider champion.

**Results:**

A total of 147 charts were included in the study. 47% of the Lund Browder charts were not revised which can be explained two ways. First, the initial TBSA was correct and confirmed by the provider champion. Second, the provider champion performed the initial TBSA estimation, and no revised chart was needed. Once the patient arrived at our hospital and a burn team member filled out the Lund Browder chart, there was a high degree of correlation between the initial TBSA and the revised TBSA performed by a provider champion. Only 6% of the patients (9 patients) had a change in the CPT code with 7 of them being upsized to the next 10%tile but no change in level of care or resuscitation parameters. 2 patients were downsized from 20-29% to 10-19% and 20-29% to < 10% TBSA. The patient with a change from 20-29% to < 10% TBSA was the only patient identified where there was a change in fluid resuscitation and level of care the patient received. We did identify that although the TBSA percentage was accurate, the actual diagram including correct location of burn and drawing on the diagram were often inappropriately identified and needed revision.

**Conclusions:**

The percentage of TBSA is an essential indicator of severity of burn guiding providers on fluid resuscitation, level of care, treatment strategies, and prognostic outcomes. We found a high degree of correlation between initial TBSA and revised TBSA estimates performed by a provider champion once the patient arrived to our ABA verified burn center. The revised Lund Browder charts were more accurate in location and completeness of the diagram and therefore still likely to be beneficial.

**Applicability of Research to Practice:**

Once the patient arrives at an ABA verified burn center, the accuracy of the initial TBSA on the Lund Browder chart highly correlates with a revised TBSA performed by a provider champion. This speaks to the education that is provided to burn team members and familiarity of burn injuries at a burn center.

**Funding for the Study:**

N/A

